# MedVantage: A Primary Care Model for Populations With High Social and Medical Needs

**DOI:** 10.31486/toj.24.0033

**Published:** 2024

**Authors:** Audrey Shawley, Sakshi Sharma, Matthew Jung, John Lim, Liam Kavanagh, Richard Li, Marshall Wadleigh, Amal Nehmeh, G. Dodd Denton, Kathy Jo Carstarphen

**Affiliations:** ^1^ The University of Queensland Medical School, Ochsner Clinical School, New Orleans, LA; ^2^ Department of Data Science, Ochsner Health System, Ochsner Clinic Foundation, New Orleans, LA; ^3^ Department of Epidemiology, Tulane University School of Public Health and Tropical Medicine, New Orleans, LA; ^4^ Department of Finance, Ochsner Clinic Foundation, New Orleans, LA; ^5^ Department of Internal Medicine, Ochsner Clinic Foundation, New Orleans, LA

**Keywords:** *Health disparate minority and vulnerable populations*, *healthcare disparities*, *primary health care*, *patient care team*, *social determinants of health*, *socioeconomic disparities in health*

## Abstract

**Background:** Despite the substantial expenditures on health care in the United States, persistent underperformance in health system metrics necessitates innovative approaches to address complex patient needs. The MedVantage Clinic in New Orleans, Louisiana, offers a regionally tailored, value-based primary care model targeting patients with high social and medical needs. This study provides an evaluation of the efficacy of the MedVantage Clinic in improving the cost of care and service utilization for this population.

**Methods:** We conducted a retrospective case-control study using data from electronic health records and claims data from 2017 to 2018. The case group was composed of patients with high social and medical needs who were enrolled in the MedVantage Clinic, and the control group consisted of propensity-matched non-MedVantage Clinic patients. Cost and utilization metrics—including per-member, per-month costs and inpatient length of stay—were analyzed using independent sample *t* tests and difference-in-difference calculations.

**Results:** The MedVantage Clinic group demonstrated a significant decrease in mean inpatient per-member, per-month cost ($4.20) compared to an increase in the control group ($280.20, *P*=0.017). Inpatient length of stay decreased by 1.7 days for MedVantage Clinic patients and increased by 8 days for control group patients (*P*=0.019). Although other metrics showed nonsignificant improvements, the MedVantage Clinic generated a total cost of care mean resource benefit of $305.44 per member, per month compared to the control group (*P*=0.112), with an estimated annual total benefit of $1,224,648 for 189 patients.

**Conclusion:** Our findings highlight the potential of the MedVantage Clinic to improve health care costs and utilization for patients with high social and medical needs. Despite the limitations of the study, including study duration and patient selection biases, the MedVantage Clinic demonstrated promise as a scalable model for addressing complex patient needs. Further research is warranted to explore long-term outcomes and implementation strategies for similar models nationwide.

## INTRODUCTION

Despite spending a much higher percentage of gross domestic product on health care compared to other high-income countries, the United States persistently underperforms on measures of health system outcomes, care access, efficiency, equity, and population health outcomes.^1-4^ These outcomes are in large part attributable to the challenge of effectively managing patients with complex, multimorbid conditions—a rapidly growing demographic with numerous barriers to receiving comprehensive, coordinated care.^5-8^ Systemic reforms aimed at not only enhancing care delivery but also ensuring that health care spending translates into better health outcomes for all Americans are urgently needed.

Conventional fee-for-service payment models reimburse for discrete services rather than incentivizing holistic, value-based care delivery.^9^ While initiatives such as the Comprehensive Primary Care Plus (2017) and Primary Care First (2021) models represent a transition toward value-based primary care, the Centers for Medicare & Medicaid Services (CMS) acknowledges that limitations still exist in managing populations with high social and medical needs.^10,11^ The recommendation for effectively addressing these limitations is to implement tailored regional approaches that incorporate strategies customized to the specific socioeconomic and health-related determinants of these communities. Such approaches should engage local stakeholders and leverage intersectoral collaborations to enhance health equity and ensure that care delivery is responsive to the unique needs of the population.^11,12^

Louisiana exemplifies such regional challenges. The state ranks last nationally for health outcomes, and the citizenry has high rates of multimorbidity, driven by factors such as low health literacy; scarce social support; poverty; and elevated exposure to violence, pollution, and incarceration.^13^ In 2013, the Robert Wood Johnson Foundation reported that a life expectancy gap of 25 years existed between the city and suburban neighborhoods in New Orleans.^14^ This confluence of medical and social complexity renders comprehensive primary care provision not only vital but also highly challenging to implement effectively.^15,16^ Vulnerable populations face persistent health disparities, and for this reason, equity must be a core tenet of any primary care model for patients with high social and medical needs.

Factors such as medical complexity, multimorbidity, low health literacy, and unmet social needs disproportionately strain older adult populations.^17^ Current incentive models, particularly performance-based models that include metrics such as hospital readmission rates, can exacerbate disparities by penalizing providers who treat patients with multiple comorbidities and chronic conditions. Desired outcomes are often difficult to achieve in high-risk patients. Providers failing to meet these stringent standards face reduced payments or increased financial risks, deterring them from caring for complex patient populations.^18^

Some studies that explored managing multimorbid, high-utilization patients prioritized cost cutting over optimizing health care quality and access,^19,20^ and the study of primary care patients at 5 US Department of Veterans Affairs medical centers excluded certain high-risk patients because of perceived challenges with behavioral change.^19^ However, identifying and addressing health-related social needs is imperative for enhancing care quality.^21^

## MEDVANTAGE CLINIC BACKGROUND AND GOALS

In response to these challenges, the MedVantage Clinic in New Orleans, Louisiana, was established as a comprehensive, regionally tailored, value-based primary care model to equitably serve patients with high social and medical needs. Piloted in 2017, the MedVantage Clinic was designed to serve as a prototype for a cost-effective clinic that aims to decrease health care expenditure while improving patient outcomes via targeted preventive interventions that holistically address both clinical and socioeconomic determinants (Appendix A). The MedVantage Clinic model aligns with ongoing policy reforms that prioritize value and quality in primary care, providing a potential solution to the limitations of traditional fee-for-service payment models and addressing the need for equity in health care.^22^

The MedVantage Clinic is located in an urban setting at Ochsner Health, a major institution that serves the Greater New Orleans area. The primary aim of the clinic is to take a multidisciplinary approach to addressing the health-related and social needs of patients. The primary target population for the clinic is elderly patients who are considered at high risk for hospital admission and readmission.

The goals of the clinic are to increase health care access, identify and address health-related social needs, use targeted interventions to improve patient adherence, implement a multidisciplinary staffing model, and serve as a training site for health professionals.

### Increase Health Care Access

The MedVantage Clinic offers same-day appointments, accommodates frequent visits, and extends appointment duration to address multiple patient concerns during each encounter. For patients who can leave their homes, the frequency of visits at the MedVantage Clinic varies from weekly to every 6 months, and visits consistently exceed 30 minutes. The extended time frame for medical consultations allows for thorough patient assessments to enhance the quality of care and reduce the need for acute care interventions.^23^

For homebound patients, the MedVantage Clinic offers home visits and telehealth appointments. The clinic collaborates with mobile palliative and chronic care nurse practitioner services to offer end-of-life care coordination.

### Identify and Address Health-Related Social Needs

Health-related social needs such as housing instability, food insecurity, and violence exposure increase utilization and worsen outcomes.^24^ Screening tools including the Patient Health Questionnaire-4, General Anxiety Disorder-7, and Single Item Literacy Screener are administered to assess mental health, health literacy, and home/food/safety needs. Positive screens prompt referrals to behavioral health, social workers, and on-site financial/pharmacy assistance services.

Transportation is a common barrier to care for the MedVantage Clinic target population. The MedVantage Clinic addressed this issue through a partnership with a rideshare service, Lyft Concierge, to provide clinic-funded transportation for patients. Rideshare services were initially coordinated by existing clinic staff and later supported by the addition of a community health worker to the team.

### Use Targeted Interventions to Improve Patient Adherence

Poor adherence to treatment plans has many root causes, including low health literacy, lack of trust in the physician, and complex instructions.^25-27^ To improve medication adherence, MedVantage Clinic staff assess patient understanding of medication regimens, discuss complex dosing schedules, and explore opportunities for appropriate deprescribing of high-risk medications. MedVantage Clinic staff use shared decision-making techniques, trauma-informed care, and transparent language to increase patient trust in the physician. MedVantage Clinic works closely with Ochsner outpatient pharmacy staff to reduce the cost pressures of medications and to increase medication accessibility for patients. This partnership highlights the vital role that pharmacists also play in addressing the social determinants of health and the needs of patients.^28^

To simplify complex dosing schedules, MedVantage Clinic pharmacists offer pill packing services to reduce patient pill burden, which can be associated with medication adherence.^29^ The packs include a key with physical descriptions of the pills, the name of each medication, and color coding that helps patients with low health literacy differentiate between morning and evening medications.^29^ Pill pack assembly occurs in the same building as the MedVantage Clinic, allowing for same-day adjustments and refills.

### Implement a Multidisciplinary Staffing Model

Given the labor-intensive nature of care required for patients with high social and medical needs, a consistent and engaged team is essential for clinical efficiency. Over time, the core clinical staff evolved to include the provider (medical doctor or nurse practitioner), care manager (registered nurse), care navigators (medical assistants), community health worker, social worker, and trainees (residents and students in medicine, nursing, public health, pharmacy, and undergraduate premedical education).

For patients with health-related social needs, the interdisciplinary team offers a diverse set of skills to provide creative and targeted interventions. Such a team-based approach has been associated with improved patient outcomes and clinician well-being.^30^ Having the multidisciplinary team physically co-located in the clinic allows for face-to-face communication, fluid collaboration, and increased collegiality.

### Serve as a Training Site for Health Professionals

The clinic serves as a clinical training hub for medical students, internal medicine residents, pharmacy students and fellows, nurse practitioner students, undergraduate premedical students, public health interns, and nursing students. Trainees are provided an immersive opportunity to explore health-related social needs under the supervision of the lead provider. A mentor model, with the trainee driving the pace of the encounter, provides an opportunity for trainees to explore the moral, social, and structural determinants of health of patients without the pressures of fee for service or volume impacting their interactions. Clinical practitioners-in-training demonstrate an increase in knowledge base when they have a supportive, collaborative relationship with a mentor and coach.^31^ This mentor model allows trainees to build confidence in their clinical interactions and reasoning, while simultaneously ensuring patient safety and optimal care.^31^

## MEDVANTAGE CLINIC VIABILITY

This study was undertaken to identify the various interventions that differentiate the MedVantage Clinic from conventional primary care models; to underscore the essential services that underpin the care of patients with complex social and medical needs; and to demonstrate the advantages of implementing this model to address health care costs and utilization.

To assess the financial viability of the MedVantage Clinic, we conducted a retrospective case-control study to compare the cost of care and utilization of patients who were managed at the MedVantage Clinic vs patients who were not managed at the MedVantage Clinic.

## METHODS

### Study Design

This retrospective, case-control study used patient data from the Ochsner Health electronic medical record (EMR) (Epic Systems Corporation) and claims data abstracted from the Milliman Health Cost Guidelines–Grouper application.^32^ We present data on cost and utilization during the MedVantage Clinic pilot year, 2017 to 2018. The cost variables (reported as per-member, per-month mean values) are total cost of care and inpatient, emergency department (ED), professional, outpatient, ancillary (including laboratory and radiology), and drug costs. In addition, we provide net margin comparisons from 2016 through 2021 for the MedVantage Clinic provider vs other primary care providers (PCPs) at Ochsner Health. The service utilization variables (reported as mean values) are inpatient length of stay (days) and inpatient and ED utilization.

This study was submitted to and approved by the Ochsner Clinic Foundation Institutional Review Board, ensuring compliance with ethical standards.

### MedVantage Group Selection

The case group were nondeceased patients who were continuously enrolled in the MedVantage Clinic for 90 days. Initial selection criteria identified 245 patients. The case group was refined based on the availability of at least 365 days (approximately 12 months) of claims data before and after the index date of their MedVantage Clinic enrollment in the years 2017 to 2018, thereby excluding 56 patients with incomplete claims data. Patient age, sex, race, and chronic medical conditions are presented in Table 1. Based on demographics, we believe this sample is representative of the greater Ochsner Health population (Appendix B), and based on the number of chronic conditions, we believe this sample is representative of the greater population in the United States.^4,33^ Of the 189 patients included in the MedVantage Clinic analysis, 172 had full risk Medicare Advantage insurance plans with Ochsner Health (Insurance Group A), and 17 had other partial risk arrangements (Insurance Group B).

**Table 1.** Demographics and Comorbidities by Group

Variable	MedVantage Clinic Group, n=189	Control Group, n=189
Age, mean years	76.3	78.4
Age range, years		
40-49	8 (4.2)	4 (2.1)
50-59	12 (6.3)	13 (6.9)
60-69	64 (33.9)	59 (31.2)
≥70	105 (55.6)	113 (59.8)
Sex		
Male	82 (43.4)	87 (46.0)
Female	107 (56.6)	102 (54.0)
Race		
African American/Black	91 (48.1)	81 (42.9)
White	97 (51.3)	104 (55.0)
Other	1 (0.5)	4 (2.1)
Number of chronic medical conditions, mean	2.3	2.7
Comorbidities		
Heart failure	46 (24.3)	103 (54.5)
Chronic kidney disease	54 (28.6)	99 (52.4)
Obesity	92 (48.7)	103 (54.5)
Type 2 diabetes with associated complications	61 (32.3)	92 (48.7)
Essential hypertension	148 (78.3)	131 (69.3)
Hyperlipidemia	40 (21.2)	40 (21.2)
Psychological conditions	28 (14.8)	25 (13.2)
Dementia	13 (6.9)	22 (11.6)

Note: Data are presented as n (%) unless otherwise indicated.

Health-related social needs and the interventions provided for the MedVantage Clinic group are presented in Table 2. The most prevalent need was medication-related (nonadherence, adjustments, polypharmacy) (61.9%, n=117), with almost every patient requiring a MedVantage Clinic intervention (61.4%, n=116). In addition, all patients with nutrition, cognition, home safety, and nursing home needs received interventions.

**Table 2.** Health-Related Social Needs and Interventions for Patients Enrolled in the MedVantage Clinic

Need/Intervention	All Patients, n=189	Male, n=82	Female, n=107
Need			
Medications^a^	117 (61.9)	51 (62.2)	66 (61.7)
Smoking, alcohol, drug abuse	73 (38.6)	38 (46.3)	35 (32.7)
Mobility^b^	93 (49.2)	38 (46.3)	55 (51.4)
Nutrition	53 (28.0)	19 (23.2)	34 (31.8)
Cognition^c^	54 (28.6)	25 (30.5)	29 (27.1)
Home safety^d^	15 (7.9)	7 (8.5)	8 (7.5)
Nursing home^e^	22 (11.6)	8 (9.8)	14 (13.1)
Sensory^f^	32 (16.9)	16 (19.5)	16 (15.0)
Intervention			
Medications	116 (61.4)	50 (61.0)	66 (61.7)
Smoking, alcohol, drug abuse	67 (35.4)	33 (40.2)	34 (31.8)
Mobility	90 (47.6)	37 (45.1)	53 (49.5)
Nutrition	53 (28.0)	19 (23.2)	34 (31.8)
Cognition	54 (28.6)	25 (30.5)	29 (27.1)
Home safety	15 (7.9)	7 (8.5)	8 (7.5)
Nursing home	22 (11.6)	8 (9.8)	14 (13.1)
Sensory	26 (13.8)	12 (14.6)	14 (13.1)

^a^Medications needs include patient nonadherence, medication adjustments, and polypharmacy.

^b^Mobility needs include the inability or difficulty to ambulate because of being bedridden or needing mobility aids (eg, walker or crutches).

^c^Cognition needs include dementia and depression.

^d^Home safety needs include domestic violence and dangerous living conditions (eg, floods, pests, infestations).

^e^Nursing home needs include problems with qualifying for certain nursing facilities or needing transfer to another facility.

^f^Sensory needs include deficits in hearing or seeing.

Notes: These data were collected for the 2017 MedVantage Clinic group via retrospective chart review in 2023. Because this information was not being systematically collected at the time of the patient encounters, we believe these numbers are artificially low. Data are presented as n (%).

### Control Group Selection

The control group was identified by propensity matching with the MedVantage Clinic group using age, sex, race, mean number of chronic conditions, cost of care, and hierarchical condition category risk adjustment factor score (Appendix B).

Hierarchical condition categories are derived from the *International Classification of Diseases, Tenth Revision* (ICD-10) codes, a medical disease classification defined by the World Health Organization. Risk adjustment is the process of adjusting payments and benchmarks to better reflect the degree of illness.^34^ The risk adjustment factor is calculated from numerous demographic variables including age, sex, living situation, and disability status. Taken together, the hierarchical condition category risk adjustment factor score allows CMS to estimate future spending and helps providers understand the health characteristics of their patient population.^34^ Higher hierarchical condition category risk adjustment factor scores identify patients with a greater than average disease burden. Lower scores correlate with healthier patients; however, low scores may falsely indicate a healthy population when chart documentation is poor or Medicare risk adjustment coding is incomplete.

Control group patients included in this study were not enrolled in Ochsner Health chronic care management programs. Demographic characteristics and comorbidities for the control group are shown in Table 1. The table shows a notable distinction between the case and control groups: a greater mean number of chronic medical conditions for the control group (2.7) vs the MedVantage Clinic group (2.3). Although social determinants of health barriers were apparent to providers placing the referrals to the MedVantage Clinic, those social determinants of health were not quantifiable in Epic EMR risk scores or hierarchical condition category capture.

### Statistical Analysis

Continuous variables were compared 1 year pre (2017) and 1 year post (2018) the index date for each group. The longitudinal changes in variables for each group were then compared against each other and reported as difference-in-difference. Propensity matching with a difference-in-difference case/control and pre/post analysis is an internally validated, standardized process used for value-based programs at Ochsner Health and has been used to evaluate other value-based programs.^35,36^ A before-and-after bivariate analysis was conducted using an independent sample *t* test to determine statistical significance (CI 95%, *P*<0.05).

### Data Sources and Validation

For the MedVantage Clinic group, costs and hierarchical condition category risk adjustment factor score data were obtained using a proprietary dashboard that is refreshed every month to track the program. Humana Medicare Advantage claims data were validated based on a Humana Medicare Advantage performance Tableau data internal dashboard that is sourced from raw claims files.^36^ The Tableau data internal dashboard was used to compile net margin and mean hierarchical condition category risk adjustment factor scores by Ochsner PCPs for years 2016 through 2021. We compared these results with those of the leading provider at the MedVantage Clinic.

## RESULTS

### Cost of Care Analysis

We found a per-member, per-month resource benefit for the fully capitated MedVantage Clinic group (Insurance Group A) of $305, for an annual total estimate of $629,520 (Table 3). Similarly, we showed that clinical documentation of hierarchical condition category risk adjustment factor score for Insurance Group A resulted in a per-member, per-month resource benefit of $266, for an annual total estimate of $549,024. These findings were calculated using a 0.35 risk score differential and geographic-specific estimates derived from the CMS ratebook.^37^ Our estimate for the total annual resource benefit generated by the MedVantage Clinic (Insurance Group A and B) was $1,224,648 for 189 patients (Table 3).

**Table 3.** Cost of Care by Group, 2017-2018, and MedVantage Clinic Cost of Care Benefit Analysis

	Control Group, n=189			MedVantage Clinic Group, n=189				
Variable	Pre-2017	Post-2018	Difference (x)	Pre-2017	Post-2018	Difference (y)	Difference-in-Difference (x–y)	*P* Value
Total cost of care	$749.75	$1,368.08	$618.33	$902.51	$1215.40	$312.89	$305.44	0.112
Clinical documentation/HCC score^a^	1.67	2.30	0.63	1.50	2.49	0.99	0.36	0.072
**Total Cost of Care Benefit for the MedVantage Clinic Group, Poststudy Period**								
**Insurance Group A: Fully Capitated Arrangements, n=172**								
Total cost of care	**Monthly**				**Annual**			
Per Group A member	$305				$3,660			
Group A total	$52,460				$629,520			
Clinical documentation/HCC RAF benefit								
Per Group A member	$266				$3,192			
Group A total	$45,752				$549,024			
Total resource benefit per member	$571				$6,852			
Total resource benefit for Group A	$98,212				$1,178,544			
**Insurance Group B: Other Partial Risk Arrangements, n=17**								
Total resource benefit per member	$226				$2,712			
Total resource benefit for Group B	$3,842				$46,104			
**Combined Panel of Insurance Groups A + B, n=189**								
Total resource benefit	$102,054				$1,224,648			

^a^The clinical documentation/HCC (hierarchical condition category) score is a decimal value derived from the HCC coding system that assesses patient complexity and predicts future health care costs based on documented diagnoses. Introduced by the Centers for Medicare & Medicaid Services in 2004, HCC coding uses *International Classification of Diseases, Tenth Revision* (ICD-10) codes to assign risk scores, incorporating demographic factors to calculate a patient's risk adjustment factor (RAF) score. The HCC reflects the expected health care resource utilization and costs, considering the severity and number of chronic conditions. HCC RAF coding aids in accurate risk adjustment for value-based payment models, ensuring fair comparisons of quality and cost metrics across patient populations.

Notes: Monetary values are in US dollars. The prestudy period (pre-2017) is defined as the period 1 year prior to the enrollment in the MedVantage Clinic. The specific approaches and resources are described in the MedVantage Clinic Background and Goals section. The poststudy period (post-2018) is defined as the period 1 year following enrollment. Data were sourced from the Ochsner Health Epic (Epic Systems Corporation) electronic medical record and Milliman Health Care Guidelines—Grouper. The table demonstrates theorized cost improvement for the total panel (n=189) based upon cost of care and assigned HCC RAF.

HCC, hierarchical condition category; RAF, risk adjustment factor.

We validated total per-member, per-month expense for 25 members’ medical record numbers in a randomly selected 25-patient spot check for the fully capitated population (Insurance Group A). One member had a total per-member, per-month expense of $114,815.06. This value is close to the external and internal expense in the Tableau data internal dashboard, which showed a total per-member, per-month expense of $114,873 for one member. This validation was observed for several other medical record numbers.

We found a significant difference in per-member, per-month cost for mean inpatient expense, with a decrease in cost for the MedVantage Clinic group of $4.20 and an increase for the control group of $280.20 (*P*=0.017) (Table 4). The difference-in-difference cost saved by the MedVantage Clinic group compared to the control group was $284.40 (Table 4).

**Table 4.** Cost of Care and Service Utilization by Group, 2017-2018

	Control Group, n=189			MedVantage Clinic Group, n=189				
Variable	Pre-2017	Post-2018	Difference (x)	Pre-2017	Post-2018	Difference (y)	Difference-in-Difference (x–y)	*P* Value
Cost, mean, per member, per month								
Total	$749.75	$1,368.08	$618.33	$902.51	$1,215.40	$312.89	$305.44	0.112
Inpatient	$256.80	$537.00	$280.20	$318.80	$314.60	–$4.20	$284.40	**0.017**
Emergency department	$15.27	$18.73	$3.46	$15.88	$17.60	$1.72	$1.74	0.680
Professional	$166.38	$283.74	$117.36	$169.21	$265.50	$96.29	$21.07	0.459
Outpatient	$191.06	$301.39	$110.33	$209.91	$290.76	$80.85	$29.48	0.704
Ancillary	$54.92	$99.90	$44.98	$85.43	$123.10	$37.67	$7.31	0.747
Drug	$80.63	$146.02	$65.39	$119.16	$221.52	$102.36	–$36.97	0.194
Service utilization, mean								
Inpatient length of stay, days	5.4	13.4	8.0	9.2	7.5	–1.7	9.7	**0.019**
Inpatient utilization	0.35	0.64	0.29	0.4	0.37	–0.03	0.32	**0.009**
Emergency department utilization	0.44	0.56	0.12	0.58	0.65	0.07	0.05	0.745

Notes: Monetary values are in US dollars. Statistically significant *P* values are in bold. The prestudy period (pre-2017) is defined as the period 1 year prior to the enrollment in the MedVantage Clinic. The specific approaches and resources are described in the MedVantage Clinic Background and Goals section. The poststudy period (post-2018) is defined as the period 1 year following the enrollment. Data were sourced from the Ochsner Health Epic (Epic Systems Corporation) electronic medical record and Milliman Health Care Guidelines—Grouper.

In terms of total costs, we found a per-member, per-month increase in cost of $312.89 for the MedVantage Clinic group vs an increase of $618.33 for the control group. The difference-in-difference for per-member, per-month cost was favorable for the MedVantage Clinic at $305.44, but the difference was not statistically significant (*P*=0.112) (Table 4).

Mean ED, professional, outpatient, and ancillary per-member, per-month costs were lower for the MedVantage Clinic group compared to the control group, but the differences were not statistically significant. Mean drug per-member, per-month costs were higher for the MedVantage Clinic group compared to the control group, but this difference was not statistically significant (Table 4).

**Figure 1.**
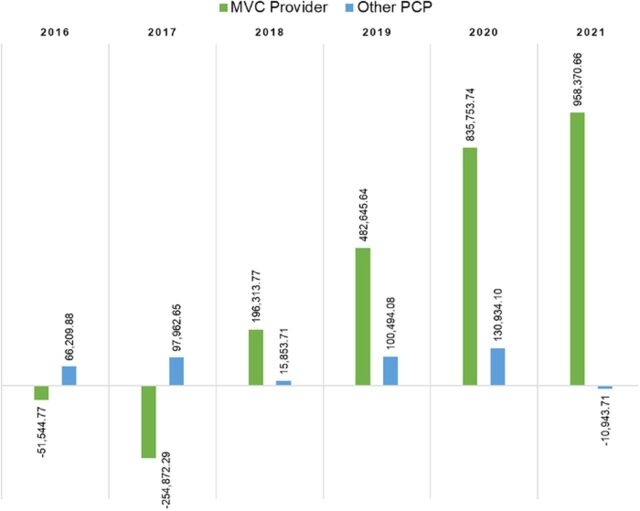
Net margin comparison in US dollars for the MedVantage Clinic (MVC) provider vs other Ochsner Health primary care providers (PCP), 2016-2021. The average net margin for all years combined was not significantly different between the MVC provider vs the other PCPs (*P*=0.306). Results were sourced from internal dashboard claims data at Ochsner Health.

The average net margin for the years 2016 through 2021 (all years combined) was high but not significantly different for the MedVantage Clinic provider compared to other PCPs (*P*=0.306) (Figure 1). The 2018 net margin was $15,853.71 for the control group and $196,313.77 for the MedVantage Clinic group, and net margins in the MedVantage Clinic group were favorable from 2018 to 2021. As the panel size increased, the net margin increased.

**Figure 2.**
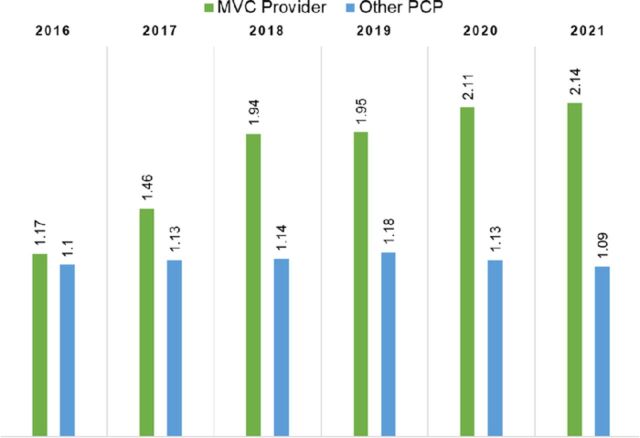
Average hierarchical condition category risk adjustment factor scores for the MedVantage Clinic (MVC) provider vs other Ochsner Health primary care providers (PCP), 2016-2021. The average hierarchical condition category risk adjustment factor score for years 2016 through 2021 (all years combined) was significantly higher for the MVC provider vs the other PCPs (*P*=0.033). Results were sourced from internal dashboard claims data at Ochsner Health.

The average hierarchical condition category risk adjustment factor score for years 2016 through 2021 (all years combined) was significantly higher for the MedVantage Clinic provider compared to other PCPs (*P*=0.033) (Figure 2). The MedVantage Clinic provider treated patients with higher average hierarchical condition category risk adjustment factor scores in all years from 2016 to 2021.

We validated average hierarchical condition category risk adjustment factor scores for 25 members’ medical record numbers in a randomly selected 25-patient spot check for the fully capitated population (Insurance Group A). One member had an average hierarchical condition category risk adjustment factor score of 4.9. This value is close to the observed value in the Tableau data internal dashboard that showed a one-member hierarchical condition category risk adjustment factor score of 4.92. This validation was observed for several other medical record numbers.

The average net margins and average hierarchical condition category risk adjustment factor scores were significantly different between the MedVantage Clinic provider and the other PCPs (*P*<0.05) for each year from 2018 through 2021, the postenrollment time frame during which the greatest benefits were expected (Figures 1 and 2).

### Service Utilization Analysis

We found a significant difference between the MedVantage Clinic group and the control group in inpatient length of stay. Length of stay increased by 8 days in the control group and decreased by 1.7 days in the MedVantage Clinic group (*P*=0.019) (Table 4). We found a similar effect in inpatient utilization, with a significant decrease in the MedVantage Clinic group vs the control group (*P*=0.009). ED utilization did not indicate a favorable change.

## DISCUSSION

The MedVantage Clinic model is focused on providing complex, multimorbid patients with resources and services to address their health needs and decrease the costs of care. Data from the clinic's first operational year demonstrated a financial benefit. The MedVantage Clinic group generated a greater total resource benefit for the health system than the matched control group (difference-in-difference of $305.44) (Table 3). While only some cost and utilization variables produced statistically significant results, all but one variable demonstrated improvements for the MedVantage Clinic group compared to the control group (Table 4). While changes in quality and the total cost of care are difficult to detect within a 5-year period,^10^ our findings indicate the value of the MedVantage Clinic model in health care delivery. We hypothesize that with a larger panel size and a longer study period, all variables would show statistical significance, as demonstrated by the increasing net margins from 2017 through 2021 (Figure 1) and the greater average hierarchical condition category risk adjustment factor scores (Figure 2) from 2016 through 2021.

Hospital utilization data revealed a mean decrease of 1.7 days in inpatient length of stay for the MedVantage Clinic group compared to an increase of 8 days for the control group, yielding a difference-in-difference of 9.7 days (*P*=0.019) (Table 4). We postulate that the comprehensive nature of the MedVantage Clinic—with interventions including a clinical home, connection with in-home care, and collaboration with end-of-life care—made this substantial impact on utilization. However, the passive intervention of time and increasing patient age may influence the progression of chronic disease in the MedVantage Clinic and control populations, leading to different hospital utilization patterns over time.

Implementation of this clinic type is scalable; however, this model cannot be offered to every patient because of high resource intensity. Institutional buy-in and a commitment to evolving organizational culture are crucial for successful implementation. Efforts to retain and support mission-driven staff are especially important during the initial stages. Challenges related to patient panel size may require expanding the complex care team to enhance care coordination, which could improve patient access to providers.

### Limitations

Several limitations should be considered in interpreting the study findings. First, the lack of financial data for the costs of providing robust MedVantage Clinic interventions, including Lyft Concierge transportation to the clinic, pill packing services, and extended appointment times, prevented a comprehensive return on investment analysis. Second, our study population consisted primarily of an older adult demographic, so extrapolating these findings to other age groups should be done cautiously. Third, despite propensity matching, differences in baseline characteristics between the case and control groups may affect the generalizability of results. Regression toward the mean should be considered as a possible cause for the observed changes in outcomes.

## CONCLUSION

Our study identifies the interventions that distinguish the MedVantage Clinic from conventional primary care models. By showcasing the effectiveness of the MedVantage Clinic in addressing the complex needs of high-risk patients, we underscore the urgent need for transformative approaches in health care delivery. The data from our pilot year demonstrate not only a financial benefit but also a tangible improvement in health care utilization. Further exploration of the relationship between such innovative clinical models and their impact on health outcomes is essential. Investing in tailored, comprehensive care for complex patients is not only a moral imperative but also a strategic investment in the sustainability and effectiveness of health care systems. Future studies should focus on understanding the long-term implications of such models and on strategies for widespread implementation to ensure equitable access to high-quality care for all patients.

## Figures and Tables

**Figure A1.**
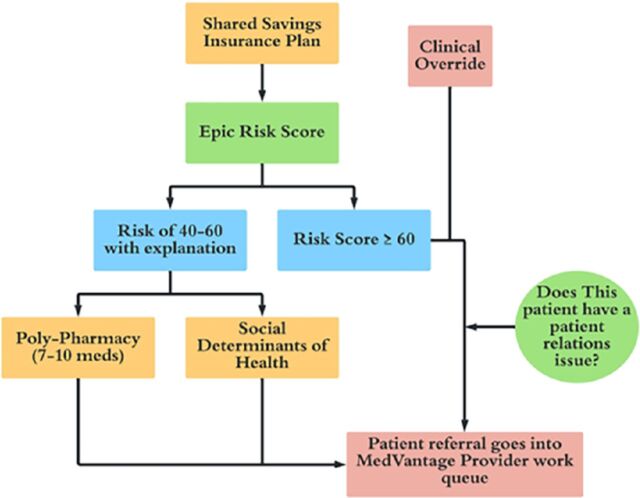
MedVantage Clinic referral process. High-risk patients were initially internally referred to the MedVantage Clinic by case managers, primary care providers, specialists, and inpatient physicians. Later, an Epic (Epic Systems Corporation) electronic medical record risk score system was implemented to identify patients likely to visit the emergency department or to be hospitalized within 1 year. The risk score incorporated variables such as the number of hospital and emergency department visits in the prior year, relationship status, whether the patient had a primary care provider, and number of chronic diseases.

**Figure A2.**
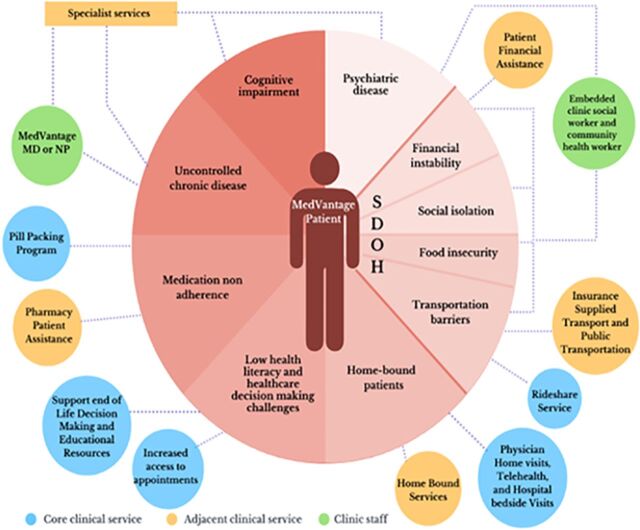
**MedVantage Clinic conceptual model. The MedVantage Clinic is built around the complex social and medical needs of high-risk patients and identifies the core clinical services provided, as well as adjacent clinical services and the clinic staff involved in providing services and interventions**. MD, medical doctor; NP, nurse practitioner; SDoH, social determinants of health.

**Figure A3.**
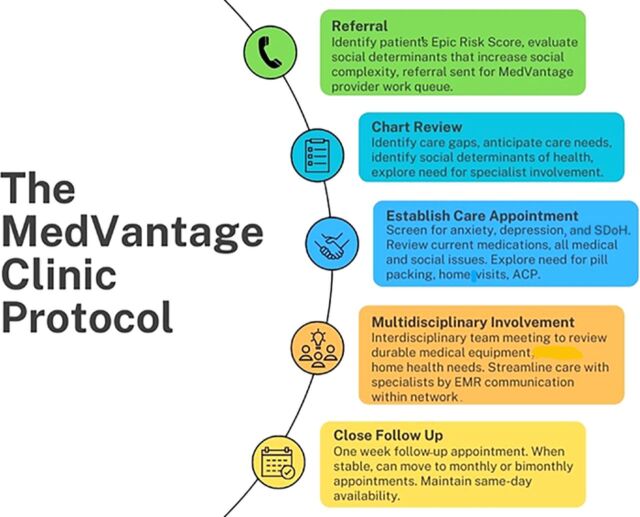
**MedVantage Clinic Protocol. MedVantage Clinic patient care begins with the referral to the clinic followed by a thorough medical record review, an initial screening appointment, identification of multidisciplinary services needs, and ongoing follow-up.** ACP, advance care planning; EMR, electronic medical record; SDoH, social determinants of health.

**Figure.**
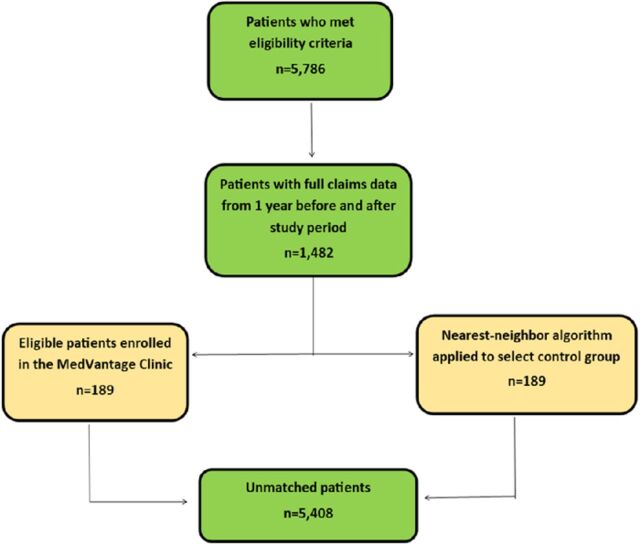
Patient selection flow chart.

## Notes

*This article meets the Accreditation Council for Graduate Medical Education and the American Board of Medical Specialties Maintenance of Certification competencies for Patient Care, Medical Knowledge, Professionalism, Systems-Based Practice.*

## Appendix B. Patient Selection and Demographic Comparison of MedVantage Clinic Group vs Unmatched Patients

**Table.** Demographics of the Matched MedVantage Clinic Group Compared to Unmatched Patients

Variable	Matched MedVantage Clinic Group, n=189	Unmatched Patients, n=5,408
Age, mean, years	78.4	74.11
Age range, years		
40-49	4 (2.1)	123 (2.3)
50-59	13 (6.9)	375 (6.9)
60-69	59 (31.2)	1,243 (23.0)
≥70	113 (59.8)	3,667 (67.8)
Sex		
Male	87 (46.0)	2,687 (49.7)
Female	102 (54.0)	2,721 (50.3)
Race		
African American/Black	81 (42.9)	3,219 (59.5)
White	104 (55.0)	1,508 (27.9)
Other	4 (2.1)	681 (12.6)
Number of chronic medical conditions, mean	2.7	9.4

Note: Data are presented as n (%) unless otherwise indicated.
